# Supplementary material for: High-Fat Foods and FODMAPs Containing Gluten Foods Primarily Contribute to Symptoms of Irritable Bowel Syndrome in Korean Adults
Source: Nutrients. 2021 Apr 15;13(4):1308. doi: 10.3390/nu13041308 (PMC8071217; doi:10.3390/nu13041308)
Supplement: Supplementary file 1 [file nutrients-13-01308-s001.zip › nutrients-1174086-supplementary.pdf]

Supplementary Table S1. Food groups of food items causing IBS symptoms.

Food Groups	Food Items
High FODMAP containing gluten	noodle, dumpling, white bread, sweet red bean bread, Sponge cake, stir-fried rice cake (tteokbokki), cornflake with milk, snack, cookies, curry rice, instant ramen, black bean sauce noodle, chinese-style noodles with vegetables and seafood, pizza, hamburger, vegetable pancake, spaghetti, wanjajeon, Korean cold noodles (naengmyeon)
High fat	instant ramen, black bean sauce noodles, chinese-style noodles with vegetables and seafoods, butter, pizza, hamburger, stock soup of bone and stew meat (Seolleongtang), pork back-bone stew (Gamjatang), loach Soup (Chueotang), beef soup, spicy sausage stew (budae jjigae), sweet and sour pork/cutlet, fried egg, pork belly, boiled pork, grilled beef, spicy pork/bulgogi/galbi, ham, stir fried chicken(chopped roast chicken), fried chicken, duck meat, fish cake, vegetable pancake, japchae, stir-fried potatoes, french fries, rice bowl, spaghetti, bowl pancake, vegetable salad
High FODMAP	multigrain rice (japgokbap), bean rice, bibimbap, miso soup, kimchi stew, kimchi stir-fried, tofu stew, tofu, bean stew(doenjang stew), pumpkin sprouts, vegetable salad, green onions, boiled broccoli, garlic, chili paste and mixed source (ssamjang), chinese cabbage kimchi (baechugimchi), other kimchi, pickles (chili, garlic, sesame leaves, onion, radish), stir-fried mushroom, steamed sweet potatoe, steamed corn, soy milk, tomato, watermelon, peach, apple, pear, persimmon, banana, kiwi, mango, dried fruit, canned fruit, carbonated drink, fruit juice, rice flour drinks, peanuts
Spicy	dongtae stew, bibimbap, stir-fried rice cakes (tteokbokki), potato soup, kimchi stew, spicy sausage stew (budaejjigae), fried chicken, cabbage kimchi(baechugimchi), other kimchi, rice bowl
Dairy	milk, liquid yogurt, yogurt, cornflake with milk, ice cream
Caffeine	coffee, green tea, chocolate, carbonated drinks (soda)
Alcohol	soju, beer, Takju (makgeolli)
